# From Viral Recovery to Autoimmunity: A Case Report of Rheumatoid Arthritis Emergence After COVID‐19

**DOI:** 10.1155/crdi/9577787

**Published:** 2026-03-10

**Authors:** Tatsuki Tsuruga, Hajime Fujimoto, Toshiyuki Ito, Atsushi Tomaru, Haruko Saiki, Taro Yasuma, Corina N. D’Alessandro-Gabazza, Esteban C. Gabazza, Tetsu Kobayashi.

**Affiliations:** ^1^ Department of Pulmonary and Critical Care Medicine, Faculty and Graduate School of Medicine, Mie University, Edobashi 2-174, Tsu, Mie, 514-8507, Japan, mie-u.ac.jp; ^2^ Department of Immunology, Faculty and Graduate School of Medicine, Mie University, Edobashi 2-174, Tsu, Mie, 514-8507, Japan, mie-u.ac.jp; ^3^ Microbiome Research Center, Mie University, Edobashi 2-174, Tsu, Mie, 514-8507, Japan, mie-u.ac.jp; ^4^ Department of Diabetes, Endocrinology and Metabolism, Faculty and Graduate School of Medicine, Mie University, Edobashi 2-174, Tsu, Mie, 514-8507, Japan, mie-u.ac.jp

**Keywords:** autoimmune disease, COVID-19, hyperinflammatory response, rheumatoid arthritis

## Abstract

A woman in her 50s contracted Coronavirus disease 2019 (COVID‐19), initially presenting with mild symptoms, and managed conservatively. However, she developed a persistent low‐grade fever and insidious joint pain for 1 month, prompting further evaluation. Chest computed tomography revealed bilateral pulmonary infiltrates, leading to hospitalization for COVID‐19‐associated pneumonia. Despite a 2‐week course of ceftriaxone and azithromycin, her condition remained unchanged. Postadmission testing revealed elevated rheumatoid factor, anti‐cyclic citrullinated peptide (CCP) antibodies, and matrix metalloproteinase‐3, suggesting an inflammatory or autoimmune process. Given concerns for immune‐mediated inflammation, she was treated with high‐dose methylprednisolone. With pneumonia improvement, she was discharged on oral prednisolone (PSL) (20 mg/day) with a planned taper. Her joint symptoms resolved, and anti‐CCP antibody levels normalized during steroid therapy. However, upon PSL tapering and discontinuation, her joint pain recurred, and anti‐CCP antibodies became positive again. A rheumatology consultation confirmed rheumatoid arthritis (RA). This case provides rare longitudinal documentation of dynamic anti‐CCP antibody changes that paralleled clinical disease activity, illustrating the progression from postviral reactive arthritis to classifiable RA. It underscores COVID‐19’s potential to trigger autoimmune dysregulation and highlights the need for long‐term follow‐up with serial autoantibody monitoring in patients with persistent musculoskeletal symptoms after infection.

## 1. Introduction

Coronavirus disease 2019 (COVID‐19) has been associated with a broad spectrum of complications, ranging from acute conditions such as acute respiratory distress syndrome and thrombosis to chronic disorders affecting the skin, gastrointestinal tract, nervous system, heart, and kidneys [1–4]. Among these complications, the development of autoimmune diseases, including rheumatoid arthritis (RA), has been reported [5]. While potential mechanisms such as molecular mimicry—where cross‐reactivity between viral antigens and self‐antigens triggers an autoimmune response—are under investigation, the precise pathophysiology remains unclear [6].

Emerging reports suggest that COVID‐19 can precipitate autoimmune phenomena, leading to the onset of inflammatory arthritis, systemic vasculitis, and connective tissue disorders [4, 7, 8]. Some patients develop transient arthritis that resolves spontaneously, while others experience persistent inflammation mimicking established autoimmune diseases [4, 7, 8]. Inflammatory markers, including rheumatoid factor (RF), anti‐cyclic citrullinated peptide (CCP) antibodies, and matrix metalloproteinases, have been detected in post‐COVID‐19 patients, suggesting a possible link between viral infection and immune dysregulation [7, 9, 10]. The impact of viral‐induced autoimmunity may be particularly significant in individuals with genetic predisposition or preexisting subclinical autoimmunity. Given the variability in disease presentation and progression, distinguishing postviral arthritis from early‐onset RA remains a clinical challenge, necessitating careful monitoring and long‐term follow‐up [11, 12].

We report a case of a patient who developed persistent inflammatory arthritis with RA–like features following COVID‐19. Laboratory findings revealed elevated RF, anti‐CCP antibodies, and matrix metalloproteinase‐3 (MMP‐3). Although corticosteroid therapy provided temporary relief, joint pain recurred after tapering, accompanied by the reappearance of anti‐CCP antibodies, leading to a diagnosis of RA. The lack of response to antibiotics, imaging findings consistent with organizing pneumonia, and relapse after corticosteroid withdrawal support a post‐COVID‐19 autoimmune etiology. This case provides rare longitudinal evidence of dynamic anti‐CCP antibody changes paralleling disease activity, illustrating progression from postviral reactive arthritis to classifiable RA and underscoring the need for continued follow‐up and serial autoantibody monitoring after COVID‐19.

## 2. Case Report

A woman in her 50s presented with fever and joint pain as her chief complaints. She had a history of mild COVID‐19 infection, which had been diagnosed by an antigen test. During the infection, she experienced fever and sore throat; although the sore throat resolved within approximately 1 week, a low‐grade fever around 37°C persisted for about 1 month until her presentation to our hospital. She had received only symptomatic treatment with antipyretics and had not undergone antiviral therapy. However, 1 month later, she developed a persistent low‐grade fever (approximately 37°C), mild dyspnea, and new‐onset joint pain, prompting her to seek medical attention. Pulmonary infiltrates were incidentally identified on computed tomography (CT) performed to investigate the persistent fever. Despite the presence of mild dyspnea, her oxygen saturation remained within the normal range, suggesting that the inflammatory involvement was limited and did not significantly impair alveolar gas exchange. She had previously completed a four‐dose COVID‐19 vaccination series.

Her past medical history was significant for rectal cancer (pTNM Stage IIIA), which had been treated with surgical resection and adjuvant chemotherapy. She had no other comorbidities and was not taking any regular medications. Her smoking history included a 20‐pack‐year history, having smoked 10 cigarettes per day from ages 20 to 40. She was employed in the food service industry. There was no family history of collagen vascular disease or psoriasis.

On physical examination, her height was 154.7 cm, weight was 47.1 kg, and body mass index (BMI) was 19.7 kg/m^2^. Her vital signs were as follows: blood pressure, 133/73 mmHg; pulse, 121 beats per minute; respiratory rate, 18 breaths per minute; temperature, 37.2°C; and oxygen saturation, 96% on room air. Pulmonary auscultation revealed coarse crackles bilaterally at the lung bases. The patient exhibited only tenderness on palpation of the distal interphalangeal (DIP) joints of both hands, the right elbow, and the left knee, with no palpable synovitis. Because this pattern of joint involvement was atypical for RA, as also noted by the rheumatology team, a diagnosis of RA was not established at the initial evaluation. No skin rashes or lesions were observed.

Laboratory findings on admission, including blood and urine tests, are summarized in Table 1. Antigen tests for COVID‐19, influenza A, and influenza B were negative. Urine antigen tests for *Streptococcus pneumoniae* and *Legionella pneumophila* were also negative. Although the serologic evaluation was not exhaustive, antinuclear antibody (ANA), myeloperoxidase‐antineutrophil cytoplasmic antibody (MPO‐ANCA), proteinase 3‐ANCA (PR3‐ANCA), and anti‐MDA‐5 antibody were all negative. In addition, no skin lesions or enthesitis suggestive of other rheumatic diseases were observed. Chest radiography at admission demonstrated decreased transparency in the right upper and lower lung fields (Figure 1(a)). Chest CT confirmed the presence of widespread, nonsegmental, predominantly peripheral pulmonary infiltrates (Figure 1(b)). Although the radiological pattern suggested possible COVID‐19 pneumonia, the presence of bilateral infiltrates with elevated inflammatory markers did not rule out community‐acquired bacterial pneumonia. Therefore, empiric antibiotic therapy with ceftriaxone and azithromycin was initiated on hospital Day 1, and the clinical response to treatment was carefully monitored. However, after 2 weeks of treatment, the patient showed no clinical improvement, with progressive pulmonary opacities (Figure 2(a)), persistent symptoms, and worsening laboratory markers. Subsequent blood tests revealed elevated levels of RF, anti‐cyclic citrullinated peptide antibodies (ACPA), and matrix MMP‐3, raising suspicion of an underlying autoimmune process. Consequently, intravenous methylprednisolone pulse therapy (1000 mg daily for three days) was initiated on hospital Day 14 due to extensive bilateral pulmonary infiltrates.

**TABLE 1:** Laboratory findings on admission.

	Values	Normal range
Hematology		
White blood cells (/μL)	11.8 × 10^3^	4000–11,000
Neutrophils (%)	80.6	40–75
Lymphocytes (%)	11	20–45
Monocytes (%)	7.6	2–10
Eosinophils (%)	0.8	1–6
Basophils (%)	0	0–2
Red blood cells (/μL)	3.56 × 10^6^	4.2–6.1 × 10^6^
Hemoglobin (g/dL)	10.5	12–18
Hematocrit (%)	31.9	35–52
Platelets	8.54 × 10^5^	1.5–4.5 × 10^5^
Erythrocyte sedimentation rate (mm/hr)	93	< 20
Biochemistry		
Albumin (g/dL)	2.8	3.5–5.5
T‐bilirubin (mg/dL)	0.6	0.1–1.2
Blood urea nitrogen (mg/dL)	8	7–20
Creatinine (mg/dL)	0.54	0.6–1.3
Sodium (mmol/L)	137	135–145
Potassium (mmol/L)	4.3	3.5–5.1
Chloride (mmol/L)	96	96–106
Aspartate aminotransferase (U/L)	22	10–40
Alanine aminotransferase (U/L)	21	7–56
Lactate dehydrogenase (U/L)	227	100–250
Glucose (mg/dL)	99	70–140
HbA1c (%)	5.4	4–5.7
Serology		
C‐reactive protein (mg/dL)	12.9	< 1
Antinuclear antibody	< 40	< 40
Rheumatoid factor (IU/mL)	319	< 15
Anticitrullinated peptide antibody (U/mL)	10.3	< 4.5
Matrix metalloproteinase‐3 (ng/mL)	71.6	< 59.7
Antiaminoacyl tRNA synthetase antibody	< 5.0	< 5.0
Antimelanoma differentiation–associated gene 5 antibody	< 32	< 32
Myeloperoxidase antineutrophil cytoplasmic antibody	< 1.0	< 3.5
Proteinase 3 antineutrophil cytoplasmic antibody	< 1.0	< 3.5
Krebs von den Lungen‐6 (U/m)	367	< 465

FIGURE 1Radiological findings on admission. (a) Plain chest X‐ray showing abnormal shadows in the right upper lung field and bilaterally in the lower lung fields. (b) Chest computed tomography (CT) on admission reveals widespread, nonsegmental infiltrative opacities predominantly in the peripheral regions of both lungs.

**Figure:**
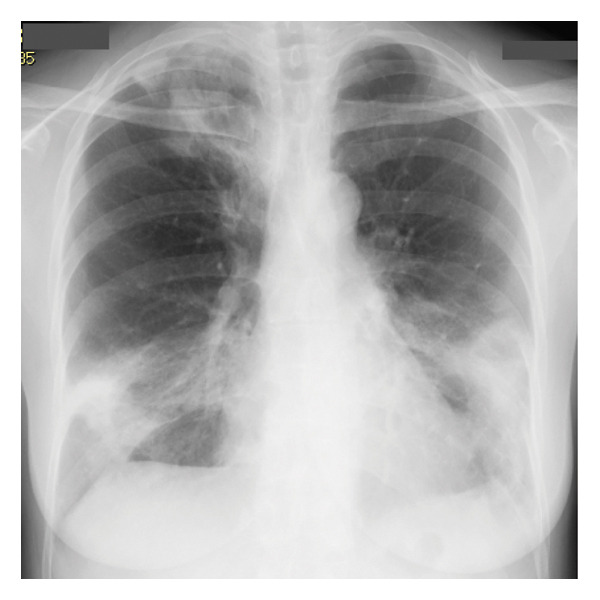
(a)

**Figure:**
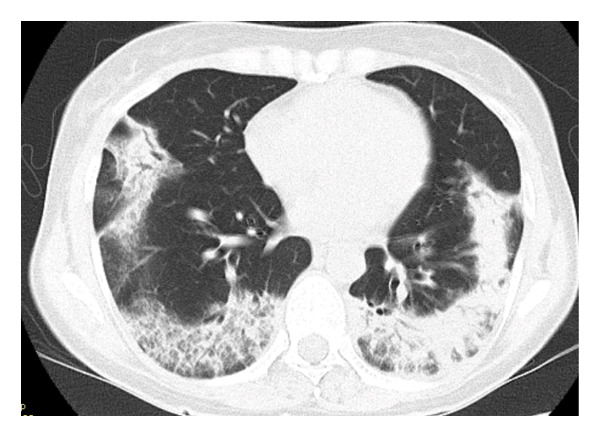
(b)

FIGURE 2Serial chest computed tomography (CT) scans. (a) CT on Day 14 of hospitalization showed progressive worsening of pulmonary opacities, with increased extent, despite antibiotic treatment. (b) CT, following steroid pulse therapy on Day 20 of hospitalization, demonstrates improved pulmonary opacities. (c) CT 4 months after treatment initiation, after steroid therapy, showing complete resolution of pulmonary opacities.

**Figure:**
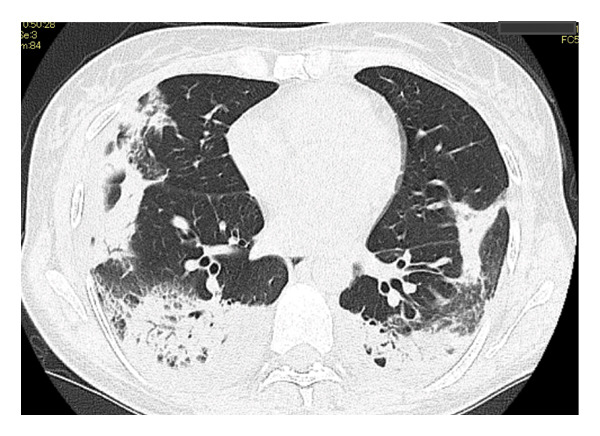
(a)

**Figure:**
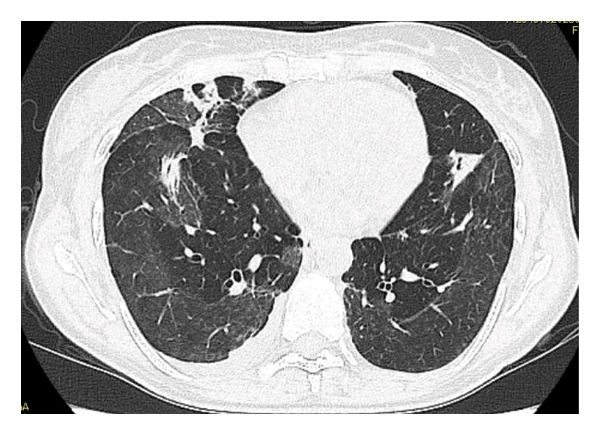
(b)

**Figure:**
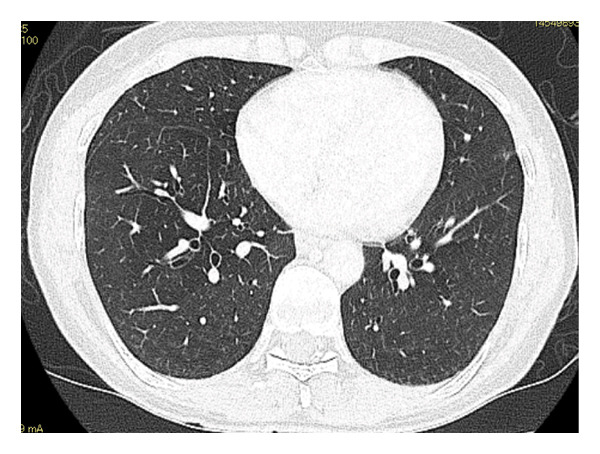
(c)

A prompt clinical response was observed (Figure 2(b)), leading to a transition to oral prednisolone (PSL) at 25 mg daily (0.5 mg/kg/day) on hospital Day 17. The patient was discharged on hospital Day 22 with a plan for gradual outpatient PSL tapering. Following steroid initiation, pulmonary imaging findings improved (Figure 2(c)), joint pain resolved, and inflammatory markers, including anti‐CCP antibody titers, normalized. PSL was tapered and ultimately discontinued after 3 months, at which time laboratory values were as follows: RF, 14 IU/mL; anti‐CCP antibodies, 3.4 U/mL; and erythrocyte sedimentation rate (ESR), 6 mm/hr.

Unfortunately, 4 months after steroid discontinuation, the patient experienced a recurrence of joint pain, accompanied by a significant increase in inflammatory markers (RF, 108 IU/mL; anti‐CCP antibodies, 9.7 U/mL; MMP‐3, 86.8 ng/mL; and ESR, 61 mm/hr). On examination, tenderness was noted in the bilateral finger joints, both elbows, the left wrist, and the right ankle. Although a rheumatology consultation had been obtained early during hospitalization to evaluate the autoantibody findings and joint symptoms, a conservative approach with observation was initially favored due to concurrent pneumonia and potential confounding factors. However, a repeat rheumatology consultation was sought in light of symptom recurrence. A musculoskeletal ultrasound revealed synovitis, which was detected in the right fourth proximal interphalangeal joint, the left first metacarpophalangeal and interphalangeal joints, the left fifth metacarpophalangeal and proximal interphalangeal joints, and the left wrist. The patient fulfilled the 2010 American College of Rheumatology/European League Against Rheumatism classification criteria for RA. A definitive diagnosis of RA was established, and treatment with PSL, sulfasalazine, and iguratimod was initiated, resulting in a favorable clinical outcome. Within approximately 2 weeks of treatment initiation, the patient showed improvement in joint symptoms although mild residual pain persisted. After about 2 months, no joint swelling or tenderness was observed, inflammatory markers had normalized, and the patient’s self‐assessment indicated good symptom control, consistent with clinical remission. The patient has since continued maintenance therapy with adjusted dosing and remains under follow‐up.

## 3. Discussion

Although COVID‐19, first identified in 2019, has become less disruptive to healthcare systems and society at large, a subset of patients continues to experience significant complications, including pneumonia, acute respiratory distress syndrome (ARDS), thromboembolism, and multiorgan dysfunction. These severe manifestations are thought to be driven, at least in part, by profound immune dysregulation characterized by excessive cytokine release and systemic inflammation in response to SARS‐CoV‐2 infection [13].

Viral infections have long been implicated in the initiation or exacerbation of autoimmune diseases. Cytomegalovirus, parvovirus B19, Epstein–Barr virus (EBV), hepatitis B and C viruses, and chikungunya virus have all been identified as environmental triggers of autoimmunity in genetically predisposed individuals [14]. Autoimmune conditions such as systemic lupus erythematosus, RA, Sjögren’s syndrome, and systemic vasculitis have been reported following viral infections, underscoring the complex interplay between viral exposure, immune activation, and loss of self‐tolerance [15].

Large population–based studies have now provided robust epidemiological evidence linking SARS‐CoV‐2 infection to an increased risk of incident autoimmune disease. A nationwide cohort study demonstrated that individuals recovering from COVID‐19 had a significantly higher incidence of newly diagnosed RA compared with matched controls, with risk increasing in proportion to infection severity [16]. Importantly, these analyses included tens to hundreds of thousands of individuals, lending substantial statistical power and minimizing reporting bias [17].

From a mechanistic perspective, immune dysregulation and autoantibody production appear to be central to SARS‐CoV‐2–associated autoimmunity. Up to 40%–50% of hospitalized COVID‐19 patients have been reported to develop at least one clinically relevant autoantibody, including RF or vasculitis‐associated antibodies [14, 18]. Elevated RF levels correlate with disease severity and systemic inflammation [19, 20], and anti‐CCP antibodies, highly specific for RA, have been detected following severe COVID‐19 even in individuals without pre‐existing rheumatic disease [21].

Multiple, nonmutually exclusive mechanisms have been proposed to explain post‐COVID‐19 autoimmune phenomena, including molecular mimicry between viral and host antigens, enhanced neutrophil extracellular trap (NET) formation, immune complex deposition, persistent viral antigen exposure, and SARS‐CoV‐2–induced alterations of the gut microbiota [15, 22–24]. Together, these processes may amplify inflammation and promote immune tolerance breach, ultimately facilitating the transition from transient autoimmunity to sustained autoimmune disease.

Several viruses, particularly EBV, have long been implicated in RA pathogenesis. EBV preferentially infects B cells, and EBV DNA, as well as latent membrane protein‐1, has been detected in RA synovial tissue, suggesting persistent immune stimulation within the joint microenvironment [25]. Notably, circulating viral nucleic acids have also been detected in a subset of patients with acute COVID‐19 [13], raising the possibility that analogous mechanisms may contribute to SARS‐CoV‐2–driven autoimmunity.

New‐onset autoimmune phenomena, including RA, have been reported not only after SARS‐CoV‐2 infection but also following COVID‐19 vaccination [26]. However, it is essential to clearly distinguish infection‐related autoimmunity from postvaccination events. SARS‐CoV‐2 infection is characterized by widespread tissue injury, high viral burden, and systemic cytokine storm, resulting in extensive autoantigen release. In contrast, vaccination typically induces a controlled and localized immune response.

Importantly, large cohort studies and meta‐analyses indicate that the absolute incidence of new autoimmune disease following COVID‐19 vaccination is extremely low, generally estimated at fewer than 5 cases per 100,000 vaccinated individuals and substantially lower than the risk associated with natural infection [27]. Several studies suggest that reported postvaccination cases may reflect unmasking of pre‐existing subclinical autoimmunity rather than de novo loss of immune tolerance [26]. Paradoxically, by reducing the severity of acute SARS‐CoV‐2 infection, vaccination may lower the long‐term risk of postinfectious autoimmune complications [27]. These findings underscore the need for balanced interpretation and caution against speculative causal attribution.

In the present case, the patient developed RA following SARS‐CoV‐2 infection, characterized by elevated RF, anti‐CCP antibodies, and systemic inflammatory markers. The absence of bacterial infection, lack of response to antibiotics, imaging findings consistent with organizing pneumonia, and relapse of arthritis following corticosteroid tapering collectively support a post‐COVID‐19 autoimmune etiology. Notably, this case provides a rare longitudinal documentation of dynamic anti‐CCP antibody fluctuations that closely paralleled clinical disease activity.

A particularly striking feature was the transient normalization of anti‐CCP titers following high‐dose corticosteroid therapy, followed by re‐emergence during dose tapering. In prospective cohorts of individuals who later develop RA, anti‐CCP antibodies typically appear years before clinical diagnosis and remain persistently positive or gradually increase over time [28]. The atypical antibody trajectory observed here suggests an acute immune perturbation rather than the classic slow preclinical evolution of RA.

This clinical course may be best interpreted within a “multihit” framework. The patient’s 20‐pack‐year smoking history—an established risk factor for anti‐CCP–positive RA through promotion of protein citrullination—may have provided an initial immunological predisposition. Subsequent SARS‐CoV‐2 infection likely acted as a potent second hit, inducing a systemic inflammatory surge sufficient to cross the threshold for sustained autoimmune disease [15].

Comparable scenarios have been described in patients with malignancy who develop autoimmune disease following additional immune stressors. For example, individuals treated with immune checkpoint inhibitors frequently develop RA‐like inflammatory arthritis, reflecting immune dysregulation in a primed host [29]. These observations suggest that underlying immune imbalance—whether due to smoking, cancer, or prior therapies—may synergize with viral infection to lower the threshold for autoimmune disease emergence.

## 4. Conclusion and Future Perspectives

This case demonstrates that SARS‐CoV‐2 infection can precipitate a rapid breakdown of immune tolerance and progression to classifiable RA, even in patients who transiently achieve serological normalization. Based on this experience, we recommend that patients with persistent musculoskeletal symptoms following COVID‐19 undergo structured follow‐up for at least 6–12 months, including serial clinical assessments and autoantibody monitoring, particularly anti‐CCP antibodies.

Early referral to rheumatology and timely diagnostic intervention are essential to prevent irreversible joint damage. At present, reliable predictors of post‐COVID‐19 autoimmune disease remain poorly defined, representing a critical gap in knowledge. Future research should focus on identifying high‐risk populations, clarifying immunological mechanisms underlying infection‐triggered autoimmunity, and establishing evidence‐based surveillance strategies. Accumulation of well‐characterized longitudinal cases will be crucial for developing personalized prevention and management approaches in the postpandemic era.

## Author Contributions

Resources: Tatsuki Tsuruga, Hajime Fujimoto, Toshiyuki Ito, Atsushi Tomaru, and Tetsu Kobayashi. Supervision: Haruko Saiki and Tatsuki Tsuruga. Visualization: Tatsuki Tsuruga, Taro Yasuma, Corina N. D’Alessandro‐Gabazza, Esteban C. Gabazza, and Tetsu Kobayashi. Writing the original draft: Hajime Fujimoto and Corina N. D’Alessandro‐Gabazza. Writing review and editing: Esteban C. Gabazza, Corina N. D’Alessandro‐Gabazza, and Tetsu Kobayashi.

## Funding

This study received no financial support.

## Disclosure

All authors approved the final version of the manuscript.

## Consent

Informed consent was obtained from the patient.

## Conflicts of Interest

The authors declare no conflicts of interest.

## Data Availability

The data that support the findings of this study are available on request from the corresponding author. The data are not publicly available due to privacy or ethical restrictions.
